# Spectrum of Kikuchi-Fujimoto Disease: A 10-Year Study in a Tertiary Care Centre in South India

**DOI:** 10.7759/cureus.69888

**Published:** 2024-09-21

**Authors:** Meryl S Kottarathil, Lawrence D'Cruze, Sandhya Sundaram, Suman H Kalantri, Akanksha Malik

**Affiliations:** 1 Pathology, Sri Ramachandra Institute of Higher Education and Research, Chennai, IND; 2 Pathology and Laboratory Medicine, Sankara Nethralaya Hospitals, Chennai, IND; 3 Pathology, Kailash Deepak Hospital, New Delhi, IND

**Keywords:** cervical lymphadenopathy, fever, kfd, kikuchi-fujimoto disease, mpo and cd68 positive histiocytes, necrotizing histiocytic lymphadenitis, young females

## Abstract

Background

Kikuchi-Fujimoto disease (KFD) is a benign lesion of the lymph nodes predominantly seen in younger women and is a condition associated with a good prognosis due to its indolent nature. Since KFD is chiefly a diagnosis of exclusion, it is liable to a higher degree of misdiagnosis.

Aim

In our study, we attempt to document and analyse the demographic, clinical, and pathological spectrum of cases diagnosed as KFD in a South Indian tertiary care centre over a period of 10 years.

Methodology

This study was conducted in a retrospective observational manner. Descriptive statistics, including simple frequency and percentage, were used to analyse and summarise the collected data.

Results

Among the 44 cases obtained, 36 patients were female and eight were males, with an age range of eight to 70 years. The mean age of the affected population was 26 years. Fever was a presenting complaint in 63.6% of cases, and 100% of the cases had lymphadenopathy, chiefly affecting the posterior cervical group of nodes (32/44 cases). Multiple lymph node groups were involved in 22 cases. Most of the nodes were larger than 1 cm in their greatest dimension. On analysis of peripheral smears, 43.2% of patients showed leukopenia. Anti-nuclear antibody (ANA) analysis showed a positive status among two patients, simultaneously establishing the diagnosis of systemic lupus erythematosus (SLE). A history of treated tuberculosis was present in four patients, and one patient had a history of non-compliance with TB treatment. Two patients had recently been diagnosed with TB and were on treatment. The involved nodes were subjected to fine-needle aspiration studies in 13 patients; 12/13 cases showed reactive lymphadenitis, while one case had atypical lymphoid cells. The involved nodes of all the patients were excised and sent for histopathological analysis. The diagnosis of KFD was confirmed on the biopsy.

Conclusion

In this study, we note the clinical presentations of all the patients diagnosed with KFD and elaborate on the investigations that were employed to verify the diagnosis via exclusion that enabled differentiation of KFD from its close mimickers, which have specific treatment. Keeping this entity in mind is essential in order to prevent overdiagnosis, as most often this condition resolves spontaneously, and in occasional cases, symptomatic treatment is sufficient for its management.

## Introduction

Described in 1972 independently by two pathologists, Dr. Kikuchi [1] and Dr. Fujimoto [2], Kikuchi-Fujimoto disease (KFD) is a self-limiting condition. Other terminologies used to describe this disease include Kikuchi-Fujimoto lymphadenopathy, Kikuchi disease, and necrotising histiocytic lymphadenitis [3]. Our understanding of this subacute necrotising lymphadenopathy seen predominantly in young, Asian women remains unclear in certain aspects, despite the numerous cases in published literature. The exact aetiology and pathogenesis of the condition are yet to be fully elucidated. Proposed mechanisms of disease pathogenesis include response to a viral infection and possible autoimmune aetiology [3,4]. Most of the patients present with fever and lymphadenopathy (commonly involving cervical nodes). Arrival at diagnosis requires a high index of suspicion and is supplemented with the assimilation of clinical, serological, and histopathological data. Kikuchi-Fujimoto disease is considered a diagnosis of exclusion when all the possible differential diagnoses have been exhausted [4]. Establishing an accurate diagnosis is of utmost importance in order to reduce the risk of overtreatment of the patient, keeping in mind the possibility of misdiagnosis [5]. Here in our study, we attempt to analyse the clinicopathological characteristics of all the cases conclusively diagnosed as KFD at our institute over a period of 10 years.

## Materials and methods

This is a retrospective observational analysis done at the Department of Pathology, Sri Ramachandra Institute of Higher Education and Research (SRIHER), Chennai, India, for a period of 10 years from June 2014 to June 2024. Ethical clearance was obtained from the Institutional Ethics Committee, SRIHER (Deemed to be University (DU)) prior to the commencement of the study (approval number: CSP-MED/22/FEB/74/25). All lymph node biopsy specimens definitively diagnosed as KFD over the period of interest were included in our study. The lymph node specimens without a primary diagnosis of KFD and core biopsies of lymph nodes were excluded from our study.

Purposive sampling technique was employed for data collection. The patients' demographic profiles, including age and gender, clinical presentation, and the investigations done, were retrieved from the hospital information system. All the histopathological case details were obtained from pathology case records. The haematoxylin and eosin (H&E) slides and immunohistochemistry (IHC) slides of all the cases selected were examined prior to inclusion in the study.

Statistical analysis

The data collected were entered into a Microsoft Excel sheet (Microsoft Corp., Redmond, WA) and analysed using IBM SPSS Statistics software, version 26 (IBM Corp., Armonk, NY). Descriptive statistics were determined by calculating simple frequency and percentage. The data analysis is presented using frequency tables.

## Results

A total of 44 cases were definitively diagnosed as KFD over the period of study. The majority of patients in our study were female (81.8%), with the most commonly affected age group being 11-25 years (50%). The mean age of the patients was approximately 26 years, with a wide observed age range, from eight years to 70 years. The demographic characteristics of the patients are summarised in Table 1.

**Table 1:** Demographic characteristics of patients with Kikuchi-Fujimoto disease Data are presented as the frequency (n) and percentage (%) of the total cases. SD: standard deviation

DEMOGRAPHIC CHARACTERISTICS		FREQUENCY (n=44)	PERCENTAGE (%)
Age (years)	< 10	2	4.5
	11-25	22	50
	26-40	16	36.4
	> 40	4	9.1
	Mean ± SD	25.97 ± 13.13	
	Range	8-70	
Gender	Male	8	18.2
	Female	36	81.8

All patients presented with lymphadenopathy (100%), which was non-tender in most cases, while four patients gave a history of painful lymphadenopathy. Among all the cases, 40 patients (90.9%) had nodes >1 cm; however, none of the nodes were larger than 3 cm.

Cervical nodes were primarily affected. Among the cervical nodes, the most common group involved was the posterior cervical group of nodes (32/44 cases), followed by the anterior cervical group (23/44 cases). Both the cervical groups were involved in 15 cases. The axillary nodes were the second most common group of nodes involved (5/44 cases), followed by nodes in other rarer regions, namely the submental, submandibular, mediastinal, and abdominal nodes. In several cases (22/44 cases), the patients presented with involvement of multiple groups of lymph nodes. A history of fever was present in 28 cases (63.6%), which was prolonged in many cases, and in a few patients, the fever showed a relapsing nature.

A peripheral smear study showed anaemia in the majority of instances. Microcytic hypochromic anaemia was seen in 12 patients (27.3%), and 13 patients (29.5%) had normocytic normochromic anaemia. Leukopenia was observed in 19 cases (43.2%). A high erythrocyte sedimentation rate (ESR) was observed in 38 cases (86.4%). A serological workup for dengue and scrub typhus was done for a few patients who presented with relapsing fever and mild thrombocytopenia. However, the tests yielded negative results, ruling out these infections.

Anti-nuclear antibody (ANA) status analysis was done for 27 patients, and it was found to be positive in two cases (4.5%) and negative in 25 cases (56.8%). The patients with positive ANA were diagnosed with systemic lupus erythematosus (SLE) along with KFD. The initial clinical and laboratory features of the cases are summarised in Table 2.

**Table 2:** Clinical and laboratory features of the participants Data are presented as the frequency (n) and percentage (%) of the total cases. TB: tuberculosis; MCHC: microcytic hypochromic; NCNC: normocytic normochromic; ESR: erythrocyte sedimentation rate; ANA: anti-nuclear antibody

S.NO	CLINICAL AND LABORATORY FEATURES		FREQUENCY (n=44)	PERCENTAGE (%)
1.	Fever	Present	28	63.6
		Absent	16	36.4
2.	History of TB	Nil	37	84. 1
		Newly diagnosed TB on treatment	2	4.5
		Non-compliant to TB treatment	1	2.3
		Treated TB	4	9.1
3.	Node size	<1 cm	4	9. 1
		>1 cm	40	90.9
4.	Peripheral smear	No anaemia	7	15.9
		MCHC anaemia	12	27.3
		NCNC anaemia	13	29.5
		Normal study	12	27.3
5.	Leukopenia	Absent	25	56.8
		Present	19	43.2
6.	ESR	High	38	86.4
		Normal	6	13.6
7.	Serology for dengue and scrub typhus	Not done	29	65.9
		Negative	15	34.1
8.	ANA status	Not done	17	38.7
		Negative	25	56.8
		Positive	2	4.5

Fine needle aspiration cytology (FNAC) of the involved nodes was done for 13 cases (29.5%); most of the cases showed a reactive picture. One case was documented to have atypical lymphoid cells. A bone marrow aspirate examination was done in 11 cases (25%), and they were all reactive. All the additional workups done for the patients and their findings are summarised in Table 3.

**Table 3:** Biopsy and additional investigations Data are presented as the frequency (n) and percentage (%) of the total cases. FNAC: fine needle aspiration cytology; IHC: immunohistochemistry

S. NO.	ADDITIONAL WORK-UP		FREQUENCY (N=44)	PERCENTAGE (%)
1.	FNAC findings	Not done	31	70.5
		Atypical lymphoid cells	1	2.3
		Reactive	12	27.2
2.	Marrow findings	Not done	33	75.0
		Reactive	11	25.0
3.	Histological features	Necrotising lymphadenitis	44	100
4.	Special stain study in biopsy	Done	19	43.2
		Not done	25	56.8
5.	IHC work-up of biopsy	Done	22	50
		Not done	22	50

**Figure 1:**
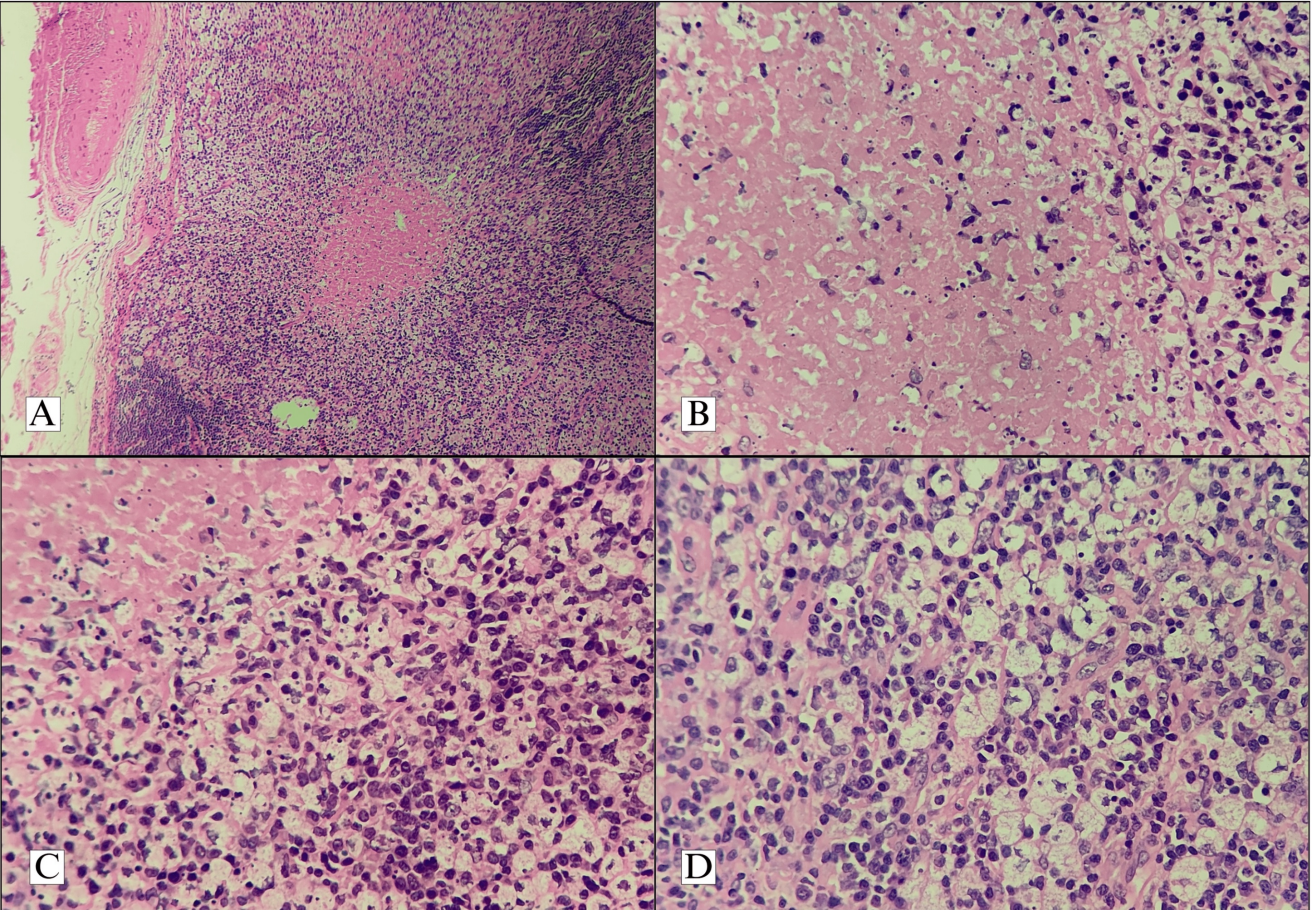
A. Low power view of an involved node with partially effaced architecture and paracortical area of necrosis (H&E, 100x); B. Higher magnification of the necrosed area showing karyorrhectic debris and lack of neutrophils (H&E, 400x); C. Interface between necrosed and viable area showing histiocytes and reactive lymphocytes (H&E, 400x); D. Foci composed predominantly of foamy histiocytes admixed with immunoblasts (H&E, 400x) H&E: haematoxylin and eosin

**Figure 2:**
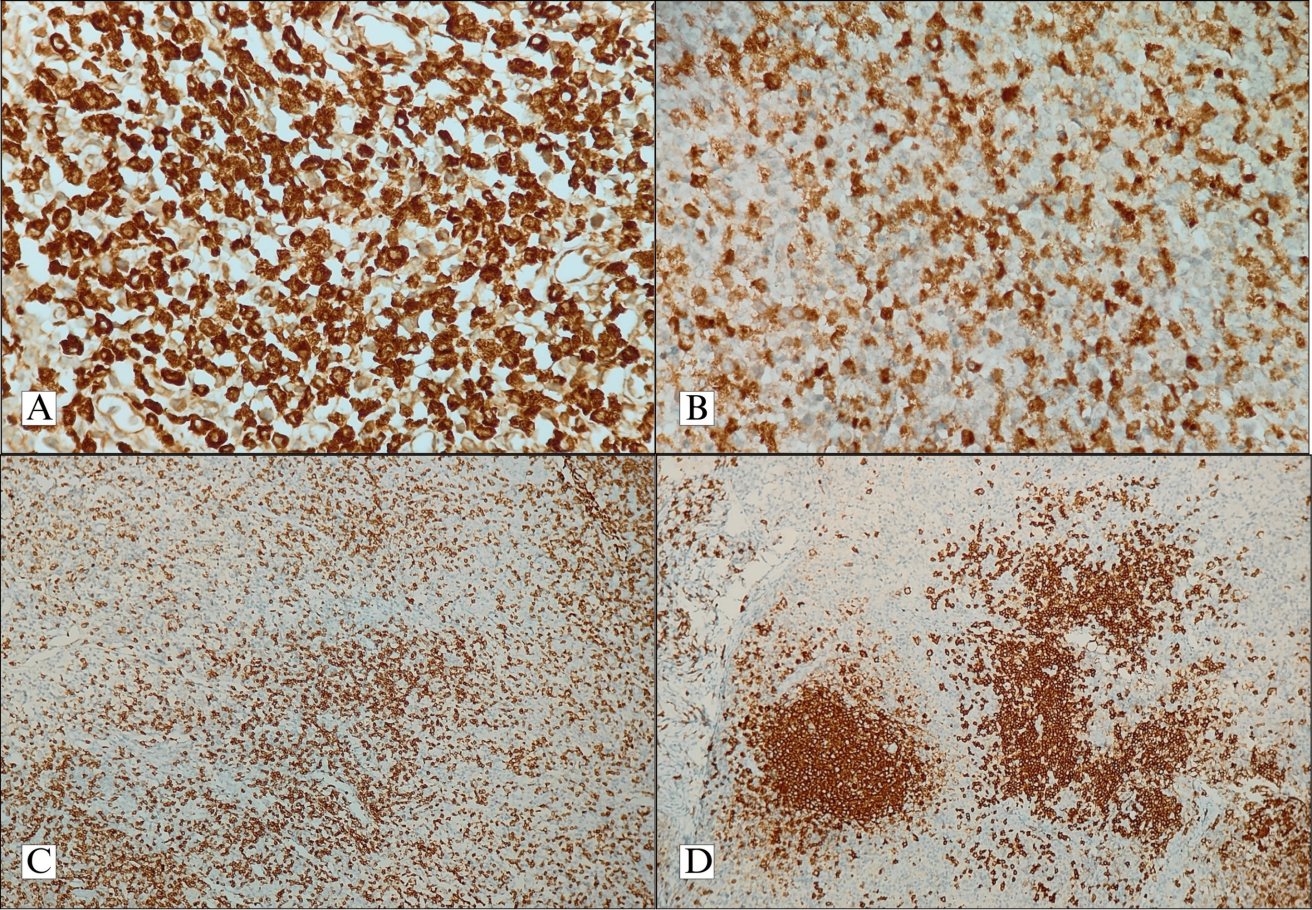
A. Histiocytes show immunoreactivity for CD68 (IHC, 400x); B. Histiocytes also show MPO positivity (IHC, 400x); C. CD3 is taken up by background reactive T lymphocytes (IHC, 400x); D. CD20 is taken up in a reactive pattern by background reactive B lymphocytes (IHC, 400x) IHC: immunohistochemistry; CD68: cluster of differentiation 68; MPO: myeloperoxidase; CD3: cluster of differentiation 3; CD20: cluster of differentiation 20

The involved nodes were excised, and histologically, all the nodes showed necrotising lymphadenitis. The nodes showed an increased presence of histiocytes and immunoblasts (Figure 1). Special stains (including acid-fast stain and stains for fungus, including periodic acid Schiff stain) were done on the biopsies of cases in which infections were a clinical differential diagnosis. The IHC work-ups were done in cases with equivocal histological pictures in order to rule out lymphoma and in certain cases to differentiate between KFD and SLE. The involved nodes showed the presence of histiocytes, which were positive for myeloperoxidase (MPO) and CD68. CD3 and CD20 were taken up by background reactive T and B lymphocytes, respectively (Figure 2).

Of 44 cases, 27 patients who were available for follow-up over a period of 12 to 16 weeks showed complete resolution of lymphadenopathy and had no other persisting symptoms. The remaining 17 patients were lost to follow-up.

## Discussion

Kikuchi-Fujimoto disease is a benign lymphadenopathy that was initially described among young women in Japan [1,2]. Initial data showed a preponderance among the Asian population, particularly young women, but as further cases were reported and published, KFD was seen to affect both genders [6,7]. In our study, 36 patients (81.8%) were female, while males constituted the remaining eight patients (18.2%). These findings are in concordance with a study published by Kucukurdali et al., documenting 77% female cases among a study population of 244 cases [8].

The most common age group affected in our study was the 11- to 25-year-old age group, with 22 cases (50%) falling in this group, immediately followed by the 26- to 40-year-old age group (16 cases, 36.4%). These findings are consistent with the findings of Bosch et al. [9]. Published literature also shows KFD cases recorded from various geographical locations; however, the Asian population seems to be affected more [4,6].

In the analysis published by Kucukurdali et al. in 2003, the most common presenting symptom of the patients was fever (35%), accompanied by joint pain and fatigue. All patients had lymphadenopathy (100%). Other clinical findings in their study included skin rash, leukopenia, and raised ESR. On further evaluation, some patients also had hepatosplenomegaly (3%) [8]. Our study showed similar findings with all the patients having lymphadenopathy (100%), and 28 patients (63.6%) presented with concurrent fever. The fever was of prolonged duration and had a relapsing nature in some patients, lasting up to three months in a few cases.

The most commonly affected group of lymph nodes involved belonged to the cervical region, among which the posterior cervical group (32/44 cases) was involved more often than the anterior cervical group (23/44 cases) of nodes. This was followed by the axillary group of nodes, which were involved in five cases. The submandibular and submental nodes were involved in a few cases. Rarer involved nodal locations recorded in our data include the mediastinal and abdominal nodes. The involved nodes were predominantly larger than 1 cm in the greatest dimension in 40 cases (90.9%); however, none of the nodes were larger than 3 cm (the largest recorded dimension of the involved nodes was 2.5 cm). The involved location of the nodes and sizes are consistent with cases published in the literature [6].

On examination of peripheral blood smears, many cases showed normocytic normochromic anaemia (13 cases, 29.5%) except in patients with long-standing febrile illness. These patients exhibited microcytic hypochromic anaemia (12 cases, 27.3%). Leukopenia was observed in 19 cases (43.2%), predominantly in patients with long-standing fever and also in one patient who had concurrent tuberculosis. The presence of mild leukopenia in patients with KFD has been documented in the literature, and our findings are in concordance with these studies [4]. Some patients also had a normal peripheral smear study (12 cases, 27.3%). Among the patients who presented with fever, two of them had recurrent episodes of fever with leukopenia and thrombocytopenia in the peripheral blood smear. Therefore, serological studies were done for dengue and scrub typhus, which turned out to be negative. Follow-up with these patients was uneventful. These cases may indicate an atypical presentation of febrile illness in KFD.

Kikuchi-Fujimoto disease is also often found to be associated with a moderately elevated ESR. Kucukurdali et al. documented that 40% of KFD cases showed elevation in ESR in their analysis [8]. In the bulk of the cases (38 cases, 86.4%) studied, the ESR numbers were moderately elevated. Three cases showed significant elevation of ESR (> 100 mm/h), with two of these cases being recently diagnosed with TB and were on treatment for the same.

Five patients had a history of tuberculosis, among whom four had undergone treatment, while one of the patients did not complete the treatment (non-compliant). Two of our cases had recently been diagnosed with tuberculosis and were on treatment for the same at the time of diagnosis of KFD. Published literature was extensively searched for patients who were diagnosed simultaneously with tuberculosis and KFD. However, such instances were scarce. A case report by Tewoldemedhin et al. documented a case of KFD that was diagnosed and had a history of treated tuberculosis. They proposed infection with *Mycobacterium tuberculosis* as a possible inciting agent for the development of KFD [10]. Lin et al. also documented the presence of a history of treated tuberculosis among five patients in a series of 61 cases of KFD [7]. Among our 44 cases, seven cases have a treated history of TB or were recently diagnosed with TB and on treatment. Yet, the lack of currently available literature indicates that further study needs to be done in order to elucidate the possible association between infection with tuberculosis and the development of KFD.

Of all the documented cases, ANA analysis was done in 27 cases in view of an equivocal diagnosis being given on histology with a differential diagnosis of KFD and SLE. Among these patients, two patients showed positivity for ANA (and were diagnosed concurrently with SLE). The diagnostic findings of Pileri et al. of CD68 and MPO IHC positivity in histiocytes in KFD were collaborated within these two cases in establishing the diagnosis of KFD [11]. These patients at follow-up had no other lymphadenopathies or recurrent fever, which they had earlier presented with. However, they had to be continued with treatment for SLE. Autoimmune aetiology is one of the proposed theories for KFD, and the presence of SLE simultaneously or diagnosed prior to, simultaneously, or after the diagnosis of KFD has been recorded in the literature, though previous research highlights that KFD has a tendency to occur prior to the diagnosis of SLE [12,13]. In these cases, the diagnosis of KFD has been established following the examination of nodes, which revealed characteristic histological findings.

Fine needle aspiration cytology of the involved lymph nodes was performed on 13 out of the 44 cases as a preliminary investigation. The FNAC results were unable to throw much light, only to the extent of finding features of reactive lymphadenitis. One case showed the presence of atypical lymphoid cells. Thus these patients were further subjected to whole node excision that revealed histological findings characteristic of KFD. According to Tong et al., a diagnosis of KFD on fine needle aspiration can be made with an accuracy of 56.25% [14]. The false negative rate in their study was as high as 50%, while the false positive rate was 37.5%. The findings to look out for in a fine needle aspirate that suggest KFD include the presence of necrosis, karyorrhectic debris admixed with prominent histiocytes, and reactive lymphocytes [15]. The role of FNAC in establishing the diagnosis of KFD remains inconclusive, and in many instances, KFD gets reported as reactive lymphadenitis on FNA. However, the role of FNAC is important in order to rule out the other important differentials of KFD, including tuberculous lymphadenitis and lymphoma [6]. Bone marrow biopsies were attempted in 11 patients with relapsing episodes of fever (pyrexia of unknown origin) and in whom TB and lymphoma were considered clinical differential diagnoses. All the patients had reactive marrow. Mild splenomegaly was identified in two patients.

All of the cases uniformly showed the presence of necrotic lymphadenitis on histological examination of the excised node. The architecture of the node was partially effaced with patchy necrosis observed mainly in the paracortical regions. Necrotic areas showed the presence of karyorrhectic debris and were characteristically devoid of neutrophils. Adjacent areas showed infiltration by histiocytes and an increased immunoblast population. Granulomas and atypical cells were not identified in the biopsies examined, and the sections also did not show the presence of extracellular haematoxylin bodies seen classically in association with lupus lymphadenitis. Among the cases in which tuberculosis or infectious aetiology was among the clinical differential diagnoses, special stains, including Ziehl-Neelsen stain and periodic acid Schiff stains, were done to rule out the presence of acid-fast bacilli and fungal organisms. The special stain studies were negative in all cases. In a few cases, in some of which SLE and lymphoma could not be ruled out on the basis of histology alone, an IHC workup was done. A panel of IHCs, which included CD45, CD3, CD20, CD68, and MPO, was done. The characteristic IHC findings of KFD include positivity of CD68 and MPO in histiocytes, which was observed in our cases. CD45 highlighted all the lymphocytes and CD3 and CD20 were taken up in a reactive pattern by the background T and B lymphocytes, respectively. Kikuchi-Fujimoto disease requires histological examination for diagnosis, and most of the cases in the literature mention the presence of partially effaced architecture with the presence of paracortical necrosis and the absence of neutrophils as findings suggestive of KFD [3,4,6]. However, there have been suggestions that KFD displays different histological appearances based on the stage of disease progression. According to Kuo, the various phases include an early proliferative phase, a late necrotic phase, and a xanthomatous phase. The early proliferative phase shows increased histiocytes and plasmacytoid dendritic cells. The presence of any extent of necrosis automatically qualifies the lesion into the necrotic phase. A preponderance of foamy histiocytes classifies the lesion to be in a xanthomatous phase [16]. However, due to the absence of data on sequential biopsy examinations done on patients diagnosed with KFD, the progression of diseases through these histological phases remains yet to be clarified [4].

Although a viral aetiology has been proposed for KFD, multiple studies done in published literature were unable to demonstrate any relationship between diagnosed cases of KFD and the presence of viral antigens [17,18]. One of the limitations in our study is that we have not done a workup for viral infections in all our cases. Immunohistochemistry for Epstein-Barr virus was done in two cases, and they were negative.

On follow-up, 27/44 cases showed complete resolution of lymphadenopathy and other symptoms over a period of three to four months. There is no targeted treatment available for KFD, and as per published literature, specific treatment is not necessary and is required only for symptomatic relief. This may include the use of analgesics and antipyretics, with steroids being reserved for severe cases that tend to recur or relapse [4,6].

Our study was limited by the fact that we only looked at cases that were conclusively diagnosed as KFD. We did not comprehensively analyse all other diagnoses of lymphadenopathy or the incidence of KFD during the study period. Furthermore, all the cases included in our study were not available for follow-up, and therefore, the course of the disease in some patients could not be verified (17/44 cases).

## Conclusions

This is a retrospective observational analysis of the spectrum of cases that were diagnosed as KFD in our institute over a period of 10 years. We have documented the demographics of the patients, how they presented clinically, and how the diagnosis was rendered. Kikuchi-Fujimoto disease remains a diagnosis of exclusion, and several investigations may be required to rule out other clinically sinister conditions with specific diagnostic tests. Fine needle aspiration cytology of the involved node is a tool that can aid in eliminating the close differentials of KFD, including tuberculosis and lymphoma. Immunohistochemistry is indispensable in cases in which lymphoma and lupus lymphadenitis are histological differential diagnoses due to the presence of CD68 and MPO-positive histiocytes in KFD. Interestingly, it was noted in our study that in a number of cases, KFD occurred in a background of treated past and present TB infections. Further research needs to be done in order to shed light on the possible relationship between these two entities. Since KFD is a self-limiting, indolent disorder with a benign course that has several potentially serious differentials, a high degree of suspicion is warranted so that patients with KFD are not misdiagnosed and subjected to overtreatment.
